# 

**Published:** 1889-06-01

**Authors:** 


### SPECIAL HOSPITAL SUNDAY NUMBER.

This Special Number will be issued on SATURDAY, JUNE 15th next, and will consist of an issue of TWENTY THOUSAND Copies. It will be sent as usual to every Clergyman and Minister of Religion who has a Collection in aid of the Metropolitan Hospital Sunday Fund, and will be distributed to the Congregations of many of the principal Churches and Chapels. Applications for Free Copies for distribution should be made at once by Clergymen and Ministers of Religion to the Publisher of THE HOSPITAL, 139, Salisbury-court, E.C., the number of copies desired being stated in the letter of application.

### NOTICE TO CORRESPONDENTS.

All MS., Letters, Books for Review, and other matters intended for the Editor, should be addressed THE EDITOR, THE LODGE, PORCHESTER SQUARE, LONDON, W.

### Notice to Advertisers and Subscribers.

All Advertisements, Orders for Copies of THE HOSPITAL, and Business Communications should be addressed to The Publisher, *not to the Editor* THE HOSPITAL, 139-140, Salisbury-court, Fleet-street, E.C.

Vol. V., now ready, 4s., post free. Cases for binding, may be had of the Publisher, at 1s. 6d. each.

### To Residents, Secretaries, and Matrons.

The Editor will be glad to have the Regulations and each Report as issued by Asylums, Hospitals, Convalescent and Nursing Institutions, as well as those of other Charities, and an early copy in duplicate of all Appeals and Festival Notices, etc., addressed to *The Editor, The Lodge, Porchester-square, London, W.* Any superintendent, secretary, matron, or other chief official who regularly sends such information may be registered, on application to the Publisher, to receive free of cost a cover for binding THE HOSPITAL in half-yearly volumes.

### Amusements and Relaxation.

### Word Competition.

Commenced April 6th, Ends June 29th.

Three Prizes of 15s., 10s., 5s., will be given for the largest number of words derived from the words set for dissection.

Proper names, abbreviations, foreign words, words of less than four letters, and repetitions are barred; plurals, and past and present participles of verbs, are allowed. Nuttall's *Standard* dictionary only to be used.

N.B.—Word dissections must be sent in WEEKLY, arranged alphabetically, with correct total affixed.

The word for dissection for this, the *Ninth* week of the quarter, being

"HERKOMER."

### Results of Seventh Week.

| Names. | May 23rd. | Totals. | Names. | May 23rd. | Totals. |
|---|---|---|---|---|---|
| Patience | 60 | 580 | Tiny | 62 | 493 |
| Nurse Duty | 62 | 560 | N'importe Qui | 52 | 457 |
| Lightowlers | 62 | 560 | Pollie | 54 | 433 |
| Scrooge | 62 | 541 | W. G. R. | 53 | 430 |
| Reldas | — | 250 | Robe | — | 150 |
| Tinie | 60 | 583 | Beryl | 39 | 391 |
| K. G. | 60 | 540 | Nesta | — | 124 |
| Broxbourne | 62 | 549 | Daffodil | — | 89 |
| Lauriston | 62 | 552 | A Schoolgirl | 49 | 192 |
| Spes | 50 | 536 | Montague | — | 79 |
| Gretta | — | 224 | Sheeny | — | 111 |
| Francesca | — | 476 | Alice R. | — | 63 |
| Heatherbell | — | 185 | Belfast | — | 163 |
| Sycamore | — | 201 | Gentian | — | 209 |
| Arctus | 48 | 469 | Hope | — | 39 |
| Amicus | 49 | 535 | Louie | — | 139 |
| Scratcher | — | 197 | Hamilton | — | 121 |
| Jemima | — | 138 | E. M. | — | 120 |
| Concours | — | 427 | F. J. D. | — | 112 |
| C. J. S. | — | 134 | Ladyr | — | 102 |
| See-Saw | 59 | 493 | Surgical Nurse | — | 128 |
| Rosemont | — | 173 | L. Pearce | — | 32 |
| Jenny Wren | 59 | 321 | Contentio | — | 98 |
| Eric | 62 | 464 | | | |

### Notice to all Correspondents.

N.B.—All letters referring to this page which do not arrive at 139 and 140, Salisbury-court, London, E.C., by the *first post on Thursdays*, and are not addressed PRIZE EDITOR, will in future be disqualified and disregarded.

### Conditions.

I. Every paper sent in must be clearly written, and one side only must be used. All competitors must mark at the head of their papers the number of their competition.

II. Each paper must be signed by the author with his or her real name and address. A *nom de plume* may be added if the writer does not desire to be referred to by us by his real name. In the case of all prize-winners, however, the real name and address will be published.

III. All communications relating to prize competitions should be addressed to "The Prize Editor, The Hospital, 139 and 140, Salisbury-court, London, E.C." Competitors are requested not to include in letters, etc., to the Prize Editor communications on matters of other business. All letters must be fully prepaid.

IV. The Prize Editor of The Hospital is the sole judge of the competitions, and his decision is in all cases final and without appeal.

V. The Prize Editor reserves the right to publish any paper sent in, whether it receives a prize or not. He does not hold himself responsible for any MSS. sent, nor can he undertake to return rejected competitions.

### Scraps and Gleanings.

Birmingham Hospital Saturday Fund has reached £6,700.

Hospital Sunday at Taunton brought in £53. A trifle less than last year.

Cholera is very bad in Ganjam. The deaths number over a thousand a week.

A new wing is about to be added to the Stockton Hospital, at a cost of £2,500.

Exeter Hospital treated 1,231 in-patients and 2,606 out-patients last year.

Lord Poltimore has been elected president of the Devon and Exeter Hospital.

Charing Cross Hospital Convalescent Home is to be built at Clacton-on-Sea.

Dewsbury Infirmary is to be congratulated on having at last freed itself from debt.

Halifax has raised £15,900 for the proposed New Infirmary. This is truly magnificent.

An anonymous donor left at the office of the London City Mission £490 in Bank of England notes.

Mr. F. Fawssett, L.R.C.P., M.R.C.S., has been appointed resident accoucheur to St. Thomas's Hospital.

At the dinner of the Poplar Hospital for Accidents, subscriptions to the amount of £1,480 10s. were announced.

Sir Morell Mackenzie has been elected an honorary member of the Medical Society of the State of New York.

The Baroness Burdett-Coutts presented the prizes to the successful exhibitors at the Cookery and Food Exhibition.

An appeal to raise an improvement fund for Queen's Hospital, Birmingham, has been issued by the committee.

Mr. Provand will call attention in the House of Commons, on June 18, to the financial position of the London hospitals.

Italy has six lady doctors: three in Bologna, one in Florence, and one in Rome, who is doctor to the Queen of Italy.

The Queen has conferred a baronetcy on Sir George Hornidge Porter, F.R.C.S.I., Surgeon-in-Ordinary to Her Majesty in Ireland.

In aid of the Orphanage of Mercy, Kilburn, a bazaar was opened in the Riding School, Knightsbridge, by the Duchess of Albany.

A grant of £500 has been promised by the authorities at the War Office towards the building fund of the Woolwich Cottage Hospital.

There is great sickness at Zanzibar; the men of the East India Squadron now at anchor there, are suffering from dysentery and fever.

On Saturday afternoon the annual prize festival of the Royal Normal College and Academy of Music for the Blind was held at the Crystal Palace.

The Queen has appointed Mr. James Reid, M.D., Physician Extra-ordinary to Her Majesty, to be one of the Physicians in Ordinary to Her Majesty.

Dr. M'Hardy, of King's College, warns the public to beware of boys playing tip-cat in the street, as the "cat" often injures the eyes of passers-by.

The German Sailors' Home in London, for the establishment of which the Baroness von Schroeder and Mrs. Lichtenberg have been very active, is now opened.

Three of Miss Ada Bell's flower pictures, now on view at Messrs. Tooths', are to be sold in aid of three hospitals in which the Princess of Wales is interested.

Sudbury has decided that they cannot raise funds to maintain the village hospitals, and prefer a free dispensary. We do not greatly admire free dispensaries.

One of the dissectors at the Paris Morgue cut off three of his fingers through letting slip a scalpel. He has been taken to a hospital, and the case is watched with interest.

An outbreak of rabies has occurred during the past week at Watford, and the justices have promptly issued a "muzzling or control" order to remain in force until Sept. 1st.

Mr. Edwin E. Jones, of Chorlton-on-Medlock Dispensary, was appointed to the post of second assistant medical officer at the Withington Workhouse, at an annual salary of £120

Drawing-room Meetings are being held in aid of the Hospital for Sick Children, Great Ormond-street. At one held at Lady Tweeddale's last week £300 was promised in the room.

Collections were made at the Parish Church, Kingston, last Sunday, in aid of the Richmond Hospital and the Surbiton Cottage Hospital. The share of the Richmond Hospital received was £11 9s. 6d.

At the Cochin Hospital, Dr. Dujardin-Beaumetz has proposed to isolate patients suffering from contagious affections in tents containing fifteen or twenty patients, and completely separated from each other.

The preliminary work towards the erection of the new Cottage Hospital for Bingley is now in progress, and the foundation-stone is to be laid on Saturday by Mr. H. Dunlop, of The Grange, Bingley.

At the festival dinner of the National Hospital for the Paralysed and Epileptic, the Lord Chancellor presided. The principal toast was proposed by the Archbishop of Canterbury, and the subscription list amounted to £2,500.

At a meeting held at the Mansion House it was resolved: "That in the opinion of the Penny a Week Collection Committee of the Hospital Saturday Fund it is desirable to secure adequate representation of the fund upon the boards of management of the various hospitals and dispensaries to which it contributes.

The Duke of Bedford has sent £50 to the fund for the restoration of St. Andrew's Church, Stockwell-green. A "Charles Dickens" bazaar will be held in Brixton Hall, Acre-lane, towards the end of next month, in aid of this fund. The chief characters in the works of the renowned novelist will be represented by the stallholders attired in appropriate costumes.

